# Mitochondrial permeability transition pore: sensitivity to opening and mechanistic dependence on substrate availability

**DOI:** 10.1038/s41598-017-10673-8

**Published:** 2017-09-05

**Authors:** Thomas Briston, Malcolm Roberts, Sian Lewis, Ben Powney, James M. Staddon, Gyorgy Szabadkai, Michael R. Duchen

**Affiliations:** 1 Neurology Innovation Centre, Hatfield Research Laboratories, Eisai Ltd., Hatfield, UK; 2 Department of Cell and Developmental Biology, Consortium for Mitochondrial Research, University College London, London, UK; 3 Department of Biomedical Sciences, University of Padua, Padua, Italy

## Abstract

Mitochondrial Ca^2+^ uptake has a key role in cellular Ca^2+^ homeostasis. Excessive matrix Ca^2+^ concentrations, especially when coincident with oxidative stress, precipitate opening of an inner mitochondrial membrane, high-conductance channel: the mitochondrial permeability transition pore (mPTP). mPTP opening has been implicated as a final cell death pathway in numerous diseases and therefore understanding conditions dictating mPTP opening is crucial for developing targeted therapies. Here, we have investigated the impact of mitochondrial metabolic state on the probability and consequences of mPTP opening. Isolated mitochondria were energised using NADH- or FADH_2_-linked substrates. The functional consequences of Ca^2+^-induced mPTP opening were assessed by Ca^2+^ retention capacity, using fluorescence-based analysis, and simultaneous measurements of mitochondrial Ca^2+^ handling, membrane potential, respiratory rate and production of reactive oxygen species (ROS). Succinate-induced, membrane potential-dependent reverse electron transfer sensitised mitochondria to mPTP opening. mPTP-induced depolarisation under succinate subsequently inhibited reverse electron transfer. Complex I-driven respiration was reduced after mPTP opening but sustained in the presence of complex II-linked substrates, consistent with inhibition of complex I-supported respiration by leakage of matrix NADH. Additionally, ROS generated at complex III did not sensitise mitochondria to mPTP opening. Thus, cellular metabolic fluxes and metabolic environment dictate mitochondrial functional response to Ca^2+^ overload.

## Introduction

Mitochondria are capable of oxidising numerous substrates based on availability and metabolic demand. The delivery of energetic substrates to mitochondria provides reducing equivalents required for serial reduction of electron transport chain (ETC) redox centres. These redox reactions are coupled to expulsion of protons from the matrix into the intermembrane space (IMS)^1^. The resulting proton electrochemical gradient (Δp), comprising a membrane potential (ΔΨ_m_) and pH gradient, is necessary for the production of adenosine triphosphate (ATP) and metabolite transport through the inner mitochondrial membrane (IMM)^2, 3^.

The functions of mitochondria extend beyond that of cellular ATP biosynthesis. Indeed, mitochondria participate in multiple regulatory signalling pathways stimulated in response to both physiological and pathophysiological stimuli. As key regulators of cell death pathways, mitochondria also play a critical role in determining cell fate^4, 5^. Thorough understanding of the (patho)physiological conditions mediating these homeostatic outcomes is important to help develop new therapeutic agents for a number of diseases including Parkinson’s Disease and stroke^6–8^.

Mitochondrial Ca^2+^ uptake plays an important role in cellular homeostasis, being driven by the maintenance of ΔΨ_m_^5, 9^. The mitochondrial permeability transition pore (mPTP) is a presumed proteinaceous entity in the IMM. Pore opening has generally been attributed to a structural change in a protein embedded within the membrane, which, under other conditions, seems to usually perform a physiological role^10, 11^. The precise molecular composition and identity of the mPTP is highly controversial but candidates include the adenine nucleotide translocase (ANT), the voltage dependent anion channel (VDAC), spastic paraplegia 7 (SPG7), phosphate carrier (PiC) and components of the ATP synthase^12–17^. Recent observations have further complicated structural understanding of the mPTP complex in that He *el al*., challenging previous claims^15, 18^ recently demonstrated intact permeability transition in the absence of membrane and C-ring subunits of the ATP synthase^19^.

To date, the only unequivocally identified component of the pore complex is cyclophilin D (CypD), a matrix cyclophilin that regulates pore opening, conferring sensitivity of the pore to inhibition by cyclosporin A (CsA)^20, 21^. Failure of cellular Ca^2+^ homeostasis and consequent mitochondrial Ca^2+^ overload is the principal trigger for mitochondrial mPTP opening^22^. High-conductance mPTP opening is associated with osmotic swelling, loss of IMM potential, uncoupling of oxidative phosphorylation and metabolic collapse^4, 22–25^.

As mPTP opening is a determinant of cell death in an ever-growing list of diseases, it is important for clinical benefit to understand pathways culminating in pore opening to identify effective interventions preventing or limiting pore opening^26–29^. To explore the impact of different metabolic conditions on the probability of pore opening, fluorescence-based analysis coupled to high-resolution respirometry was used to simultaneously measure mitochondrial Ca^2+^ uptake, ΔΨ_m_, reactive oxygen species (ROS) production and respiration. The threshold of mPTP opening was assessed under distinct metabolic conditions by measuring the Ca^2+^ buffering capacity of isolated mitochondria. Characterisation of the relationship between substrate utilisation, oxygen consumption and sensitivity to pore opening will reveal events that precede and follow mPTP opening. Here, we report variation in mitochondrial sensitivity to Ca^2+^-induced mPTP opening based on the source of electron flux through the respiratory chain, as driven by different metabolic substrates, identifying mechanisms by which energetic state determines the mitochondrial response to Ca^2+^ overload.

## Results

### Influence of mitochondrial metabolic substrate availability on mPTP opening

Mitochondria are capable of metabolising several different metabolic substrates. To understand how substrate availability determines the probability of mPTP opening, a Ca^2+^ retention capacity protocol adapted to simultaneously monitor multiple parameters was established using the FLIPR^TETRA^ high content kinetic assay system. Using this assay, mPTP opening was identified as the release of mitochondrially sequestered Ca^2+^, the failure of mitochondrial Ca^2+^ uptake (measured using the low affinity calcium indicator Fluo-4FF) and the collapse of the mitochondrial membrane potential (measured using TMRM).

**Figure 1:**
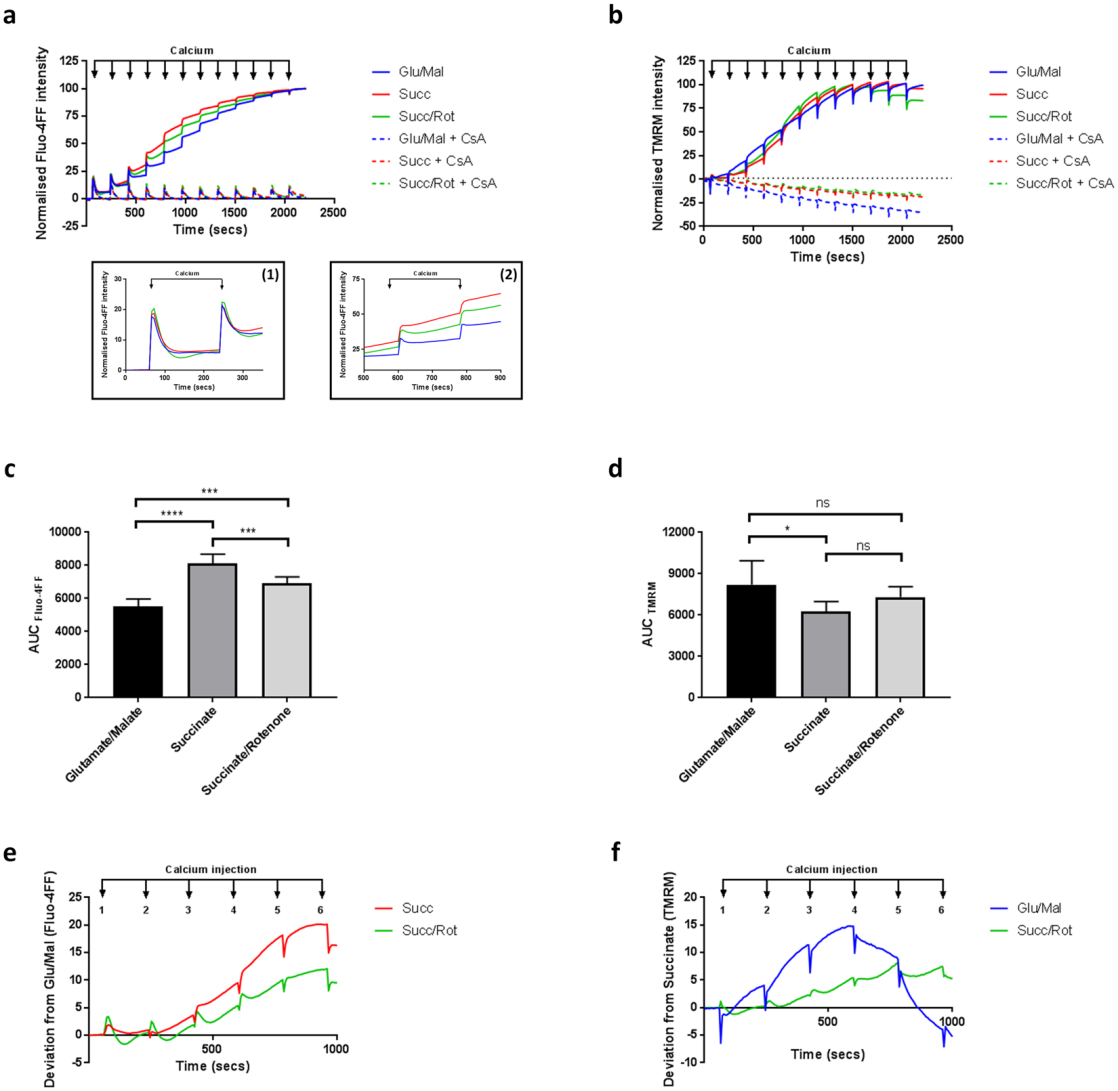
Mitochondrial metabolic substrate determines sensitivity to Ca^2+^-induced mPTP opening. Mitochondria (1 mg protein ml^−1^) were incubated with Fluo-4FF (0.35 μM) or TMRM (2 μM) in the presence of either glutamate/malate (10 mM/2 mM), succinate (10 mM) or succinate/rotenone (10 mM/1 μM). Pulses of CaCl_2_ (10 μM) were added sequentially and fluorescence measured. (**a**) Trace of extracellular Ca^2+^ fluorescence (using Fluo-4FF) and Ca^2+^ uptake in the presence different metabolic substrates prior to mPTP opening. Data was normalised using baseline and maximal Fluo-4FF fluorescence. (**b**) Trace of TMRM fluorescence and time-dependent transmembrane depolarisation after Ca^2+^ overloading and mPTP opening using different metabolic substrates. Data was normalised to baseline and maximal TMRM fluorescence (maximal depolarisation/maximal TMRM de-quench). (**c**) Area under the curve using Fluo-4FF calculated between CaCl_2_ injection numbers 4 and 5. Data were analysed using one-way ANOVA corrected for multiple comparisons using Holm-Sidak method. (**d**) Area under the curve using TMRM, calculated between CaCl_2_ injection numbers 4 and 5. Data were analysed using one-way ANOVA, corrected for multiple comparisons using Holm-Sidak method. Data are expressed as means with error bars indicating standard deviation of 6 independent experiments. (**e**) Fluo-4FF trace from (**a**), showing deviation of response as a function of glutamate/malate and (**f**) TMRM trace from (**b**) showing deviation of response as a function of succinate. ns; *P* > 0.05, *P = 0.01–0.05, **P = 0.001–0.01, ***P < 0.001. Abbreviations: ns; not significant, CsA; cyclosporin A, TMRM; tetramethylrhodamine methylester, a.u; arbitrary unit, AUC; area under the curve.

Mitochondrial suspensions were energised using mechanistically distinct substrate combinations: (1) glutamate/malate, (2) succinate or (3) succinate/rotenone. Under all metabolic conditions, initial aliquots of added Ca^2+^ (10 µM) were effectively removed from the extra-mitochondrial solution by mitochondrial Ca^2+^ uptake (Fig. 1a: insert 1) with no measurable effect on ΔΨ_m_ (Fig. 1b). Greater Ca^2+^ loads (concentrations above a threshold) caused mPTP opening and simultaneous collapse of ΔΨ_m_ under all metabolic conditions (Fig. 1a: insert 2 and Fig. 1b). However, strikingly, mitochondria oxidising glutamate/malate tolerated significantly greater Ca^2+^ loads, compared to those oxidising succinate and succinate/rotenone (Fig. 1a and c) which began to deviate from the glutamate/malate condition following 3–4 injections of calcium (Fig. 1e). A decrease of ΔΨ_m_ occurred significantly earlier in mitochondria energised with glutamate/malate compared to those metabolising succinate (Fig. 1b and d): deviation from the succinate condition occurred following just 1–2 calcium additions and prior to overt mPTP opening (as judged by Ca^2+^ retention assay; Fig. 1f). Addition of the complex I inhibitor rotenone to succinate-energised mitochondria significantly delayed mPTP opening over controls (Fig. 1c). Cyclosporin A (CsA) is a potent inhibitor of cyclophilin D (CypD), the well-established blocker of mPTP opening^30^. Under all metabolic conditions tested, mPTP opening was delayed by CsA and ΔΨ_m_ was maintained (Fig. 1a,b, Supplementary Figure 1). Independent of the metabolic substrate employed, CsA caused a dose-dependent delay in mPTP opening. IC_50_ values for CsA between substrates ranged from 86–92 nM (Table 1 and Supplementary Figure 1). Together, these data suggest that mitochondrial substrate utilisation influences the threshold required for Ca^2+^ to induce mPTP opening and has no effect of CsA potency of action.

**Table 1:** IC_50_ values for CsA in mitochondrial Ca^2+^ retention assay with different metabolic substrates.

CsA IC_50_ (M) (±95% CI)		
**Glu/Mal**	**Succ**	**Succ/Rot**
**8.6E-08** (6.5E-08 to 1.1E-07)	**9.2E-08** (8.0E-08 to 1.1E-07)	**9.0E-08** (6.8E-08 to 1.2E-07)

### Effect of metabolic substrate availability on bioenergetic consequences of mPTP opening

We next aimed to understand the consequences of the loss of mitochondrial inner membrane integrity on respiratory chain electron flow and to explore how metabolic substrate selectivity influences the bioenergetic consequences of mPTP opening. Simultaneous recordings of respiration, ΔΨ_m_ and Ca^2+^ uptake were performed in isolated mitochondria in response to Ca^2+^ overload, using the Oroboros Oxygraph 2 K high resolution respirometry system coupled with a fluorescence detection unit.

**Figure 2:**
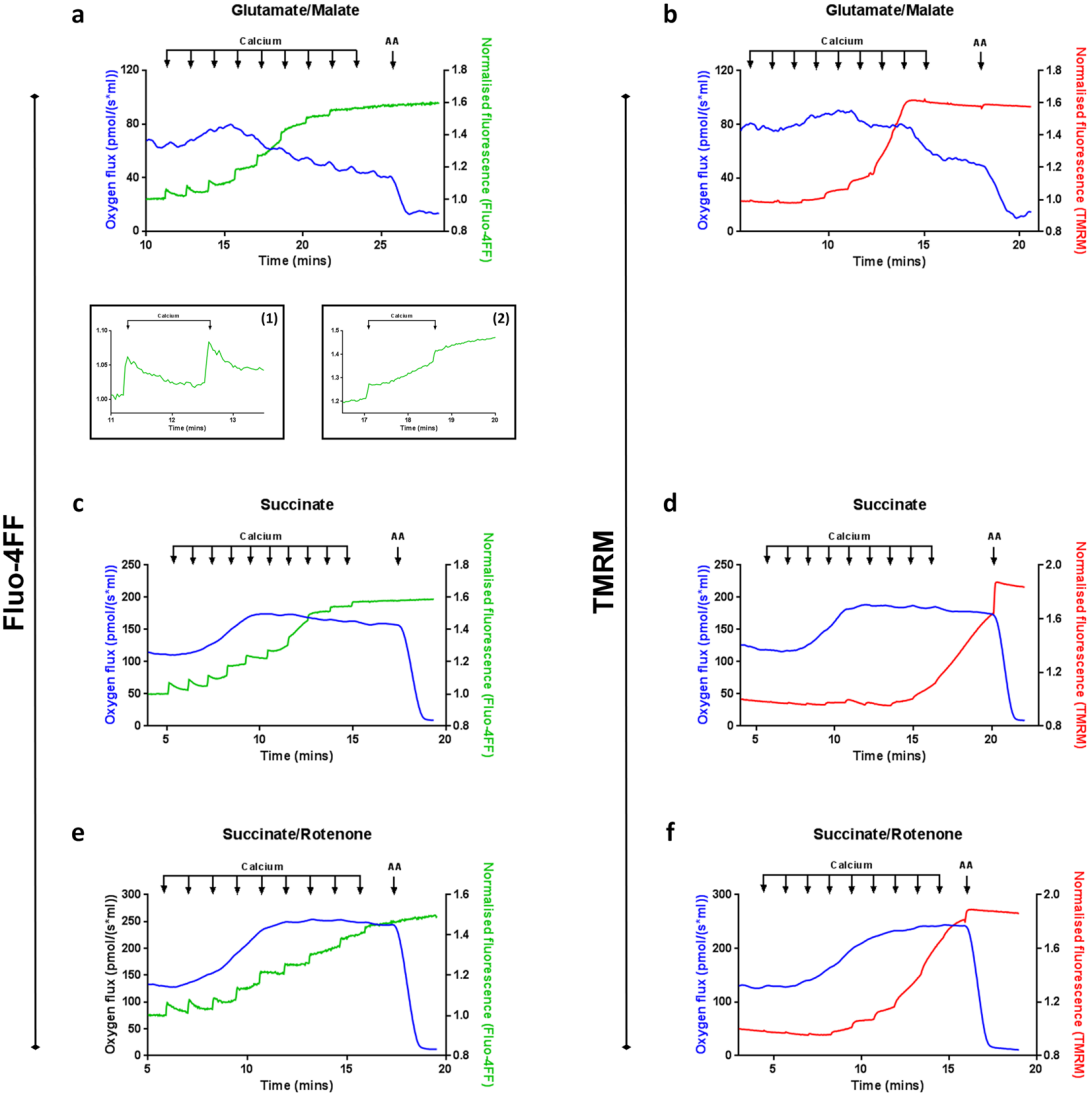
Mitochondrial metabolic substrate determines bioenergetic response to Ca^2+^-induced mPTP opening. Representative oxygen flux recording using closed-chamber, high-resolution respirometry. Mitochondria (1 mg protein ml^−1^) were maintained in the presence of either (**a**,**b**) glutamate/malate (10 mM/2 mM), (**c**,**d**) succinate (10 mM) or (**e**,**f**) succinate/rotenone (10 mM/1 μM) under constant stirring. Sequential additions of CaCl_2_ (2.5 μM) were added as indicated to induce mPTP opening. Oxygen consumption (blue trace) and extra-mitochondrial Ca^2+^ fluorescence (**a**,**c**,**e**) Fluo-4FF; green trace) or ΔΨ_m_ (**b**,**d**,**f**): TMRM; red trace) were measured in parallel using the Oxygraph 2 K equipped with fluorimeter and fluorescent control unit (Oroboros Instruments, Innsbruck, Austria). Antimycin A (2.5 μM) was used to completely inhibit respiration. Traces are representative of at least 3 independent experiments. Abbreviations: TMRM; tetramethylrhodamine methylester, AA; antimycin A.

Independent of respiratory substrate, additions of sub-threshold Ca^2+^ concentrations resulted in complete mitochondrial Ca^2+^ uptake (Green traces; Fig. 2a: insert-1, Fig. 2c,e). Prior to mPTP opening, additions of Ca^2+^induced an increase in respiration in mitochondria oxidising any of the substrates (Blue traces; Fig. 2a–f). Importantly, upon mPTP opening (i.e. the point at which Fluo-4FF fluorescence failed to return to baseline, (Fig. 2a: insert-2), the respiratory rate supported by NADH-linked substrates glutamate/malate decreased (Fig. 2a,b), while FADH_2_-driven mitochondrial respiration remained at an elevated plateau (Fig. 2c–f).

TMRM was used to assess changes in ΔΨ_m_ in parallel to measurement of respiration. In glutamate/malate energised mitochondria, transmembrane depolarisation in response to Ca^2+^ additions coincided with decreased respiration (Fig. 2b). Inhibition at cytochrome *bc*_1_ of mitochondrial complex III using antimycin A had no further effect on the TMRM signal, suggesting that the membrane was already completely depolarised (Red trace; Fig. 2b). In contrast, using the FADH_2_-linked substrate succinate (±rotenone), ΔΨ_m_ loss occurred after a Ca^2+^-induced increase in respiration, remaining sustained at an elevated respiratory rate under these conditions (see above, Fig. 2c–f). Together, these data suggest that the mitochondrial respiratory response and the kinetics of ΔΨ_m_ loss in response to mPTP opening alter depending on metabolic substrate availability. Transient stimulation of respiration was followed by decreased respiration using NADH-linked substrates while, in contrast, oxidation of FADH_2_-linked substrates resulted in a large Ca^2+^-induced increase in mitochondrial oxygen consumption, sustained during mPTP opening and consistent with both an uncoupling response and a slower rate of mitochondrial depolarisation.

### Respiratory consequences of mitochondrial Ca^2+^ load are primarily driven by mPTP

**Figure 3:**
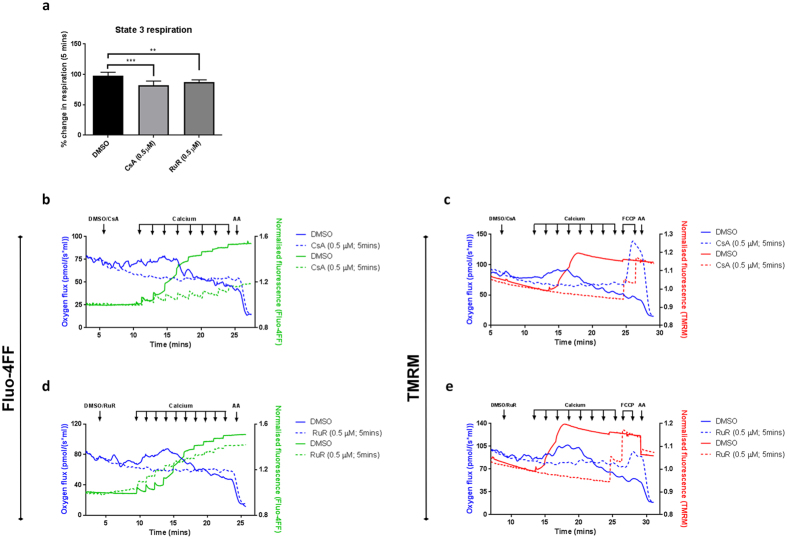
Respiratory consequences of mPTP opening are sensitive to mPTP inhibitors. Representative oxygen flux recording using closed-chamber, high-resolution respirometry. (**a**) Mitochondria (1 mg protein ml^−1^) were maintained in the presence of glutamate/malate (10 mM/2 mM) under constant stirring. CsA (0.5 μM) and RuR (0.5 μM) were added after baseline stabilisation. Percentage change in respiration was calculated after 5 minutes compound incubation. Data are expressed as means with error bars indicating standard deviation of at least three independent experiments. Data was analysed using one-way ANOVA, corrected for multiple comparisons using Holm-Sidak method. (**b**–**e**) Mitochondria were incubated as above and parallel measurements of oxygen consumption (blue trace) and either extra-mitochondrial Ca^2+^ (Fluo-4FF; green trace) or membrane potential (TMRM; red trace)were measured using the Oxygraph 2 K equipped with fluorimeter and fluorescent control unit (Oroboros Instruments, Innsbruck, Austria). Mitochondria were pre-treated with (**b**,**c**) CsA (0.5 μM) or (**d**,**e**) RuR (0.5 μM) prior to sequential additions of CaCl_2_ (2.5 μM). Abbreviations: Mito; mitochondria, TMRM; tetramethylrhodamine methylester, NADH; nicotinamide adenine dinucleotide, Succ; succinate, AA; antimycin A.

To investigate whether the effects of increasing mitochondrial Ca^2+^ load on mitochondrial respiration were triggered directly by Ca^2+^ or indirectly by mPTP opening, we used either ruthenium red (RuR) or CsA to, respectively, block mitochondrial Ca^2+^ uptake or delay mPTP opening. Mitochondria were incubated with glutamate/malate and subjected to a Ca^2+^ retention capacity assay. Consistent with published data^31–33^, we observed a small but significant decrease in State 3 respiration using both CsA and RuR (Fig. 3a). Acute incubation with CsA delayed mPTP opening, mitochondria tolerated greater Ca^2+^ loads (Fig. 3b) and both the associated decline in respiration and collapse of ΔΨ_m_ were prevented (Fig. 3b,c). Importantly, inhibition of mitochondrial Ca^2+^ uptake by RuR had the same effect on both respiration and ΔΨ_m_ (Fig. 3d,e), confirming the dependence of mPTP opening on mitochondrial Ca^2+^ uptake. Also, the Ca^2+^-induced increase in respiration prior to mPTP opening was completely prevented not only by blocking mitochondrial Ca^2+^ uptake (RuR) but also by preventing mPTP opening via CypD inhibition (CsA; Fig. 3b–e). This suggested the increase in respiration was due to Ca^2+^-induced, CsA-sensitive inner membrane uncoupling which could, however, at least to the point of mPTP opening, be compensated for through increased respiration.

### Loss of complex I-driven respiration after mPTP opening can be rescued through addition of exogenous NADH or succinate

**Figure 4:**
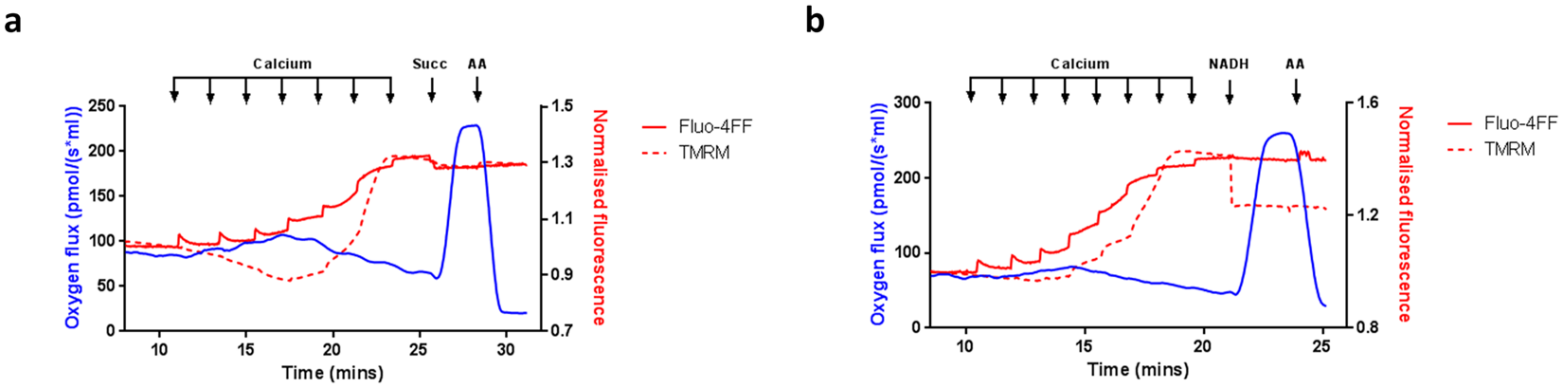
Respiratory consequences of mPTP opening are rescued by exogenous NADH or succinate. (**a**) Mitochondria (1 mg protein ml^−1^) were maintained in the presence of glutamate/malate (10 mM/2 mM) under constant stirring and subject to a Ca^2+^ retention challenge. Following pore opening, NADH (3 mM) or (**b**) succinate (10 mM) were added as indicated and respiration (blue trace), Ca^2+^ uptake (solid red trace) and ΔΨ_m_ (dashed red trace) measured simultaneously. Antimycin A (2.5 μM) was used to inhibit all respiration. Traces are representative of at least 3 independent experiments. Abbreviations: TMRM; tetramethylrhodamine methylester, NADH; nicotinamide adenine dinucleotide, Succ; succinate, AA; antimycin A.

Next we explored the mechanism behind the respiratory deficit after mPTP opening in NADH-driven respiration. Addition of exogenous succinate following mPTP opening in glutamate/malate energised mitochondria caused a rapid increase in antimycin A-sensitive respiration without affecting TMRM or extra-mitochondrial [Ca^2+^] (Fig. 4a). Addition of exogenous NADH also significantly increased the respiratory rate (Fig. 4b). Together, these data suggest that either feeding electrons into complex II (bypassing complex I) or adding saturating concentrations of NADH support respiration following mPTP opening. These findings confirm the respiratory deficits are a consequence of the loss of soluble, diffusible reducing equivalents - primarily NADH, from the tricarboxylic acid (TCA) cycle via mPTP opening, while the provision of reducing equivalents at complex II, or addition of exogenous NADH are sufficient to maintain respiratory capacity.

### Substrate selectively dictates rates of free radical generation both before and after mPTP opening

Mitochondria are an important source of cellular ROS, also implicated in sensitising mPTP formation to Ca^2+^ ^10, 34^. Thus in order to dissect the relationship between metabolic substrates, ROS generation, respiratory chain activity, mitochondrial Ca^2+^ load and mPTP opening, we measured ROS generation before and after Ca^2+^ induced mPTP opening. The primary radical species generated by mitochondria is superoxide^35–37^. Although superoxide cannot cross membranes, it is rapidly converted to H_2_O_2_ by matrix-localised superoxide dismutase (MnSOD) or spontaneously dismutates at a high rate. Additionally, H_2_O_2_ can be directly produced by some mitochondrial sites (i.e. flavins^38^). Therefore, we measured H_2_O_2_ generated by isolated mitochondria to assess overall rates of mitochondrial ROS (mtROS) generation.

**Figure 5:**
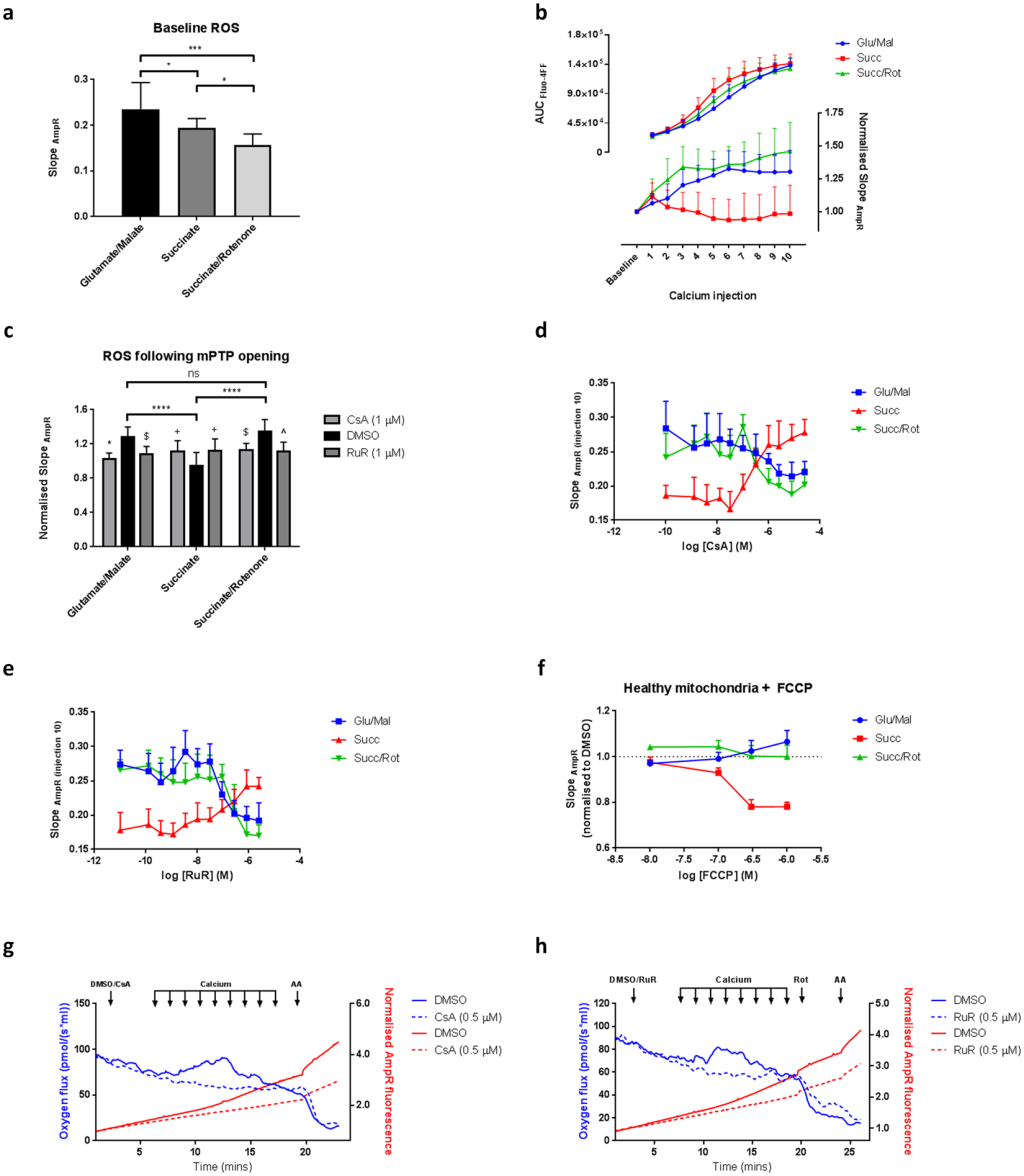
Metabolic substrate determines mitochondrial Ca^2+^-induced H_2_O_2_ production. Mitochondria (1 mg protein ml^−1^) were incubated with AmpR/HRP (10 μM/1 U ml^−1^) or Fluo-4FF (0.35 μM) in the presence of either glutamate/malate (10 mM/2 mM), succinate (10 mM) or succinate/rotenone (10 mM/1 μM). (**a**) Baseline rates of mtROS production under distinct metabolic conditions were measured using the FLIPR^TETRA^. Data was analysed using one-way ANOVA, corrected for multiple comparisons using Holm-Sidak method. (**b**) Pulses of CaCl_2_ (10 μM) were added sequentially and extra-mitochondria Ca^2+^ (Fluo-4FF; solid trace) and H_2_O_2_ (AmpR; dashed trace) recorded in parallel. Area under the curve (Fluo-4FF) and slope (AmpR) were calculated between each CaCl_2_ injection in mitochondrial energised using defined substrates. (**c**) H_2_O_2_ production calculated between CaCl_2_ injections 5–6 in the presence and absence of CsA (1 μM) or RuR (1 μM) to inhibit mPTP opening and Ca^2+^ uptake respectively. Data are normalised to baseline and are expressed as means with error bars indicating standard deviation of at least three independent experiments. Data was analysed by two-way ANOVA, corrected for multiple comparisons using Holm-Sidak method. Comparisons between groups: ns; *P* > 0.05, *P = 0.01–0.05, **P = 0.001–0.01 ***P < 0.001. Comparisons within groups: No symbol *P* > 0.05, ^+^ *P* = 0.01–0.05, ^$^ *P* = 0.001–0.01 ^^^ *P* = 0.0001–0.001, *P < 0.0001. (**d**,**e**) Mitochondria (1 mg protein ml^−1^) were incubated as above in the presence of either glutamate/malate (10 mM/2 mM), succinate (10 mM) or succinate/rotenone (10 mM/1 μM). Sequential additions of CaCl_2_ (10 μM) were added as indicated. Mitochondria were incubated in the presence of (**d**) CsA and (**e**) RuR in a 2-fold dilution series under distinct metabolic conditions. H_2_O_2_ production was measured following CaCl_2_ injection 10. Data are presented as change in AmpR fluorescence over time (slope). (**f**) Mitochondria (1 mg protein ml^−1^) were incubated with AmpR/HRP (10 μM/1 U ml^−1^) in the presence of either glutamate/malate (10 mM/2 mM), succinate (10 mM) or succinate/rotenone (10 mM/1 μM). FCCP was added for 10 minutes and fluorescence measured. Data are presented as change in AmpR fluorescence over time (slope) normalised to data in the presence of DMSO alone. (**g**,**h**) Representative oxygen flux recording using closed-chamber, high-resolution respirometry. Mitochondria (1 mg protein ml^−1^) were maintained in the presence of glutamate/malate (10 mM/2 mM) under constant stirring. Sequential additions of CaCl_2_ (2.5 μM) were added as indicated to induce mPTP opening. Oxygen consumption (blue trace) and H_2_O_2_ production (AmpR; red trace) were recorded in parallel using the Oxygraph 2 K equipped with fluorimeter and fluorescent control unit (Oroboros Instruments, Innsbruck, Austria), in the presence of (**g**) CsA (0.5 μM) and (**h**) RuR (0.5 μM; dashed traces) to inhibit mPTP opening and Ca^2+^ uptake respectively. Abbreviations: Mito; mitochondria, CsA; cyclosporin A, RuR; ruthenium red, AmpR; Amplex Red, AA; antimycin A, Succ; succinate, Rot; rotenone, a.u; arbitrary unit, AUC; area under the curve, Glu; glutamate, Mal; malate.

Extra-mitochondrial Ca^2+^ concentration (Fluo-4FF) and H_2_O_2_ production (Amplex Red; AmpR) were measured simultaneously in a Ca^2+^ retention assay protocol. The rate of baseline H_2_O_2_ production by mitochondria energised using glutamate/malate was significantly higher than that generated by succinate alone (Fig. 5a). Rotenone significantly decreased succinate-driven mtROS production, suggesting that H_2_O_2_ production in the presence of succinate originates from superoxide generated by reverse electron transfer (RET) from complex II to complex I^39–41^. Overall, however, the contribution of RET to the total AmpR signal was low (~10%), and the resulting feed forward rates of mtROS flux in succinate/rotenone-driven respiration were significantly lower than in mitochondria energised by glutamate/malate (Fig. 5a), indicating that NADH-driven forward electron flow through complexes I and III are significant sources of mtROS generation. Importantly, no relationship between rates of mtROS production and Ca^2+^ sensitivity of mPTP opening was observed when supported by complex I-linked substrates, as higher rates of mtROS generation supported by glutamate/malate-driven respiration (Fig. 5a) were not associated with a higher probability of Ca^2+^ induced mPTP opening (see Fig. 1a). However, in contrast, the reduction in baseline mtROS production by the addition of rotenone under succinate-driven respiration (Fig. 5a) reduced the probability of mPTP opening (see Fig. 1a). These results suggest that reverse electron flow through complex I contributes significantly to sensitisation of mPTP opening, while forward electron flow through complexes I and III does not affect the process.

In both glutamate/malate- and succinate/rotenone-energised mitochondria, mPTP opening was preceded by a Ca^2+^-induced increase in H_2_O_2_ production (Fig. 5b). This accompanied the Ca^2+^-dependent increased respiration (forward electron flow) which was attributed to Ca^2+^-mediated, CsA-sensitive transient uncoupling (see above). In contrast, although a Ca^2+^-induced sustained increase in respiratory rate was supported by succinate (see Fig. 1c,d), this was accompanied by only a short transient increase in H_2_O_2_ production which preceded mPTP opening (Fig. 5b). Importantly, CsA and RuR inhibited mtROS production in a dose-dependent manner with glutamate/malate and succinate/rotenone as substrates, while both inhibitors increased rates of mtROS production when mitochondria were respiring only on succinate (Fig. 5c,d,e and Supplementary Figure 2). These data suggest that the loss of ΔΨ_m_ due to mPTP opening has a differential impact on the forward (glutamate/malate and succinate/rotenone) and reverse (succinate alone) electron flows. Indeed, in healthy mitochondria (before mPTP opening), the rate of H_2_O_2_ production following uncoupling with FCCP was: (i) dose-dependently reduced when mitochondria were respiring on succinate alone and; (ii) dose-dependently increased when using glutamate/malate as substrates (Fig. 5f), confirming the differential ΔΨ_m_ dependence of mtROS production.

To further establish the relationship between mtROS production and respiration, the rates of oxygen consumption, Ca^2+^ uptake and H_2_O_2_ production were measured simultaneously using NADH-linked substrates. The Ca^2+^-induced transient increase in respiratory rate preceding permeability transition correlated with an increased AmpR fluorescence (Fig. 5g and h), and both CsA and RuR prevented the Ca^2+^-induced respiratory changes, maintaining the rates of H_2_O_2_ production at baseline levels (Fig. 5g and h).

**Figure 6:**
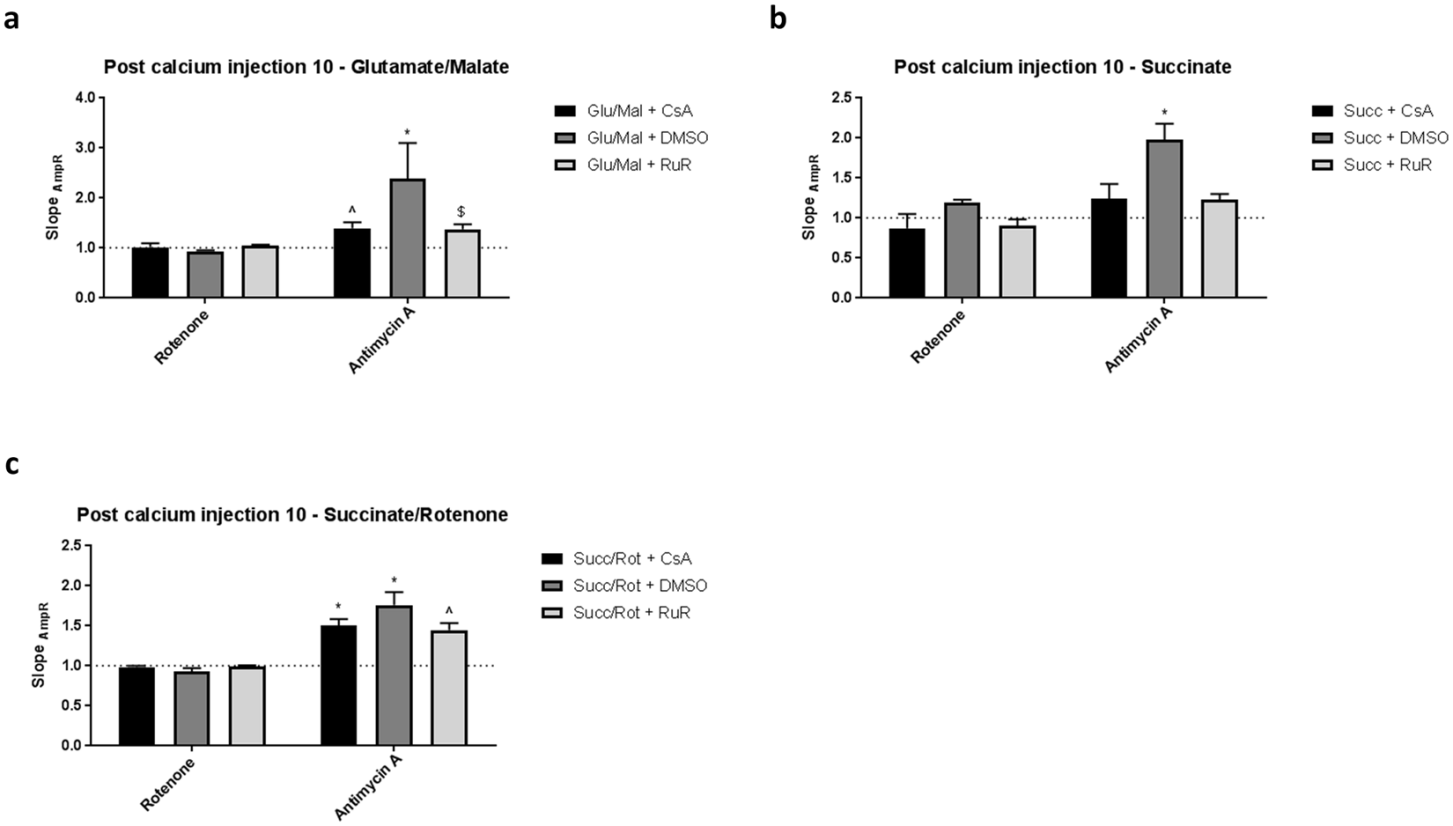
Mitochondrial metabolic substrate determines H_2_O_2_ production in response to mitochondrially-active compounds. Mitochondria (1 mg protein ml^−1^) were incubated with AmpR/HRP (10 μM/1 U ml^−1^) in the presence of either (**A**) glutamate/malate (10 mM/2 mM), (**B**) succinate (10 mM) or (**C**) succinate/rotenone (10 mM/1 μM). Mitochondria were subject to Ca^2+^ retention assay and 10 pulses of CaCl_2_ (10 μM) were added sequentially over time. H_2_O_2_ production was measured following the addition of mitochondrially-active compounds. Mitochondrially-active compounds were incubated for 10 minutes and fluorescence measured. CsA (1 μM) and RuR (1 μM) were included to inhibit mPTP opening and Ca^2+^ uptake respectively. Data are expressed as means with error bars indicating standard deviation of at least three independent experiments, normalised to vehicle (DMSO). Data was analysed using two-way ANOVA, corrected for multiple comparisons using Holm-Sidak method. No symbol *P* > 0.05, ^+^ *P* = 0.01–0.05, ^$^ *P* = 0.001–0.01, ^^^ *P* = 0.0001–0.001, *P < 0.0001. Abbreviations: CsA; cyclosporin A, RuR; ruthenium red, Glu; glutamate, Mal; malate, Succ; succinate, Rot; rotenone, AmpR; Amplex Red.

Finally, in order to verify the sites of mtROS production by the respiratory chain, the rates of H_2_O_2_ production were compared across conditions following mPTP opening (i.e. after 10 sequential CaCl_2_ additions) and complex III inhibition on the FLIPR^TETRA^ high content kinetic assay system. Antimycin A stimulated a large CsA- and RuR-sensitive increase in H_2_O_2_ production across all substrates (Fig. 6a–c). These responses were greater than those observed in control mitochondria (without Ca^2+^ stimulation), suggesting that rates of mtROS production from complex III are augmented by mPTP opening (Supplementary Figure 2). In contrast, rotenone did not increase H_2_O_2_ production in the presence of any tested substrate in this study (Fig. 6a–c), arguing against significant mtROS generation by forward flow through complex I. Taken together, these data show that respiratory substrates, by sustaining reverse or forward electron flow, determine the consequences of Ca^2+^-induced changes in mtROS production and the relationship between ΔΨ_m_ and respiratory rate ultimately determines the rate of mtROS generation.

## Discussion

We have investigated the effects of mitochondrial substrate availability on the probability of Ca^2+^-induced mPTP opening and its consequences. We observed different changes in mitochondrial respiration, membrane potential and free radical generation as a function of Ca^2+^ loading and mPTP opening under distinct metabolic conditions. In particular, the simultaneous measurements of combinations of respiratory rate, Ca^2+^ uptake, IMM potential and mtROS generation have generated new insights into the specific relationships between thee variables. Our key findings were: (i) the probability of mPTP opening is increased when respiration is supported by succinate compared to glutamate and malate, a phenomenon reduced by the presence of rotenone; (ii) the efficiency of succinate to induce mPTP opening correlates with succinate-induced RET through complex I and associated mtROS production, (iii) mPTP-induced depolarisation inhibits RET from succinate energised mitochondria; (iv) in the presence of succinate, respiration is maintained following mPTP opening and loss of mitochondrial membrane potential; (v) when respiration is supported by glutamate/malate, mPTP opening causes a transient increase in respiration followed by a decrease that can be restored by addition of NADH or succinate, consistent with respiratory inhibition due to the loss of matrix NADH; and (vi) although ROS are generated from complex III in the presence of both glutamate/malate and succinate/rotenone, this does not sensitise mPTP opening.

Previously, Huang *et al*. demonstrated that mPTP opening and subsequent cytochrome c release are more sensitive to Ca^2+^ when mitochondria are oxidising FADH_2_-linked rather than NADH-linked substrates^42^. We have added to these observations to understand metabolic consequence of Ca^2+^-induced mPTP opening, investigating bioenergetic changes, mitochondrial Ca^2+^ uptake, ΔΨ_m_ and mtROS generation. We have confirmed that respiration supported by succinate sensitises mitochondria to Ca^2+^-induced mPTP opening. Inhibition of electron flux from complex I to ubiquinone (UQ) by rotenone decreased both baseline mtROS production and the efficiency of succinate-energised mitochondria to undergo mPTP opening. This suggests that ROS generation through complex I may define the sensitivity of mitochondria to Ca^2+^-induced mPTP opening under these conditions. It is noteworthy that reperfusion following ischemia (high ΔΨ_m_, reduced UQ), has been associated with succinate accumulation and increased ROS generation by RET^39, 40, 43^. Prevention of Ca^2+^-induced RET using rotenone reduces oxidative stress in succinate-energised mitochondria, likely being responsible for the decreased mitochondrial sensitivity to Ca^2+^-induced mPTP opening. In agreement with Chouchani *et al*. reverse flow of electrons through complex I contribute to mPTP opening^43^. Additionally, we have also determined that ROS production through reverse flow is membrane potential-dependent and thus must precede mPTP opening.

We observed only a transient increase in Ca^2+^-induced mtROS production in succinate-energised mitochondria. Since high ΔΨ_m_ is necessary to drive RET and the reduction of NAD^+^, the observed Ca^2+^-induced decrease in mtROS production following mPTP opening and membrane depolarisation is likely due to the cessation of RET^41, 44–46^. Consistent with these observations, addition of the ETC uncoupler, FCCP to intact/healthy mitochondria also dose-dependently decreased mtROS production in mitochondria energised by succinate alone. In succinate-energised mitochondria, the FCCP-mediated decrease in mtROS production was observed at concentrations consistent with membrane depolarisation and increased oxygen consumption^47^, responses analogous to mPTP opening. Additionally, inhibition of mPTP opening, using CsA and RuR, prevented the Ca^2+^-induced decrease in mtROS, suggesting that mPTP opening following Ca^2+^ uptake contributes to decreased oxidative stress in mitochondria metabolising succinate alone. mPTP-induced depolarisation therefore inhibits RET and mtROS generation from succinate (similar to FCCP), correlating with a transient increase in ROS in ‘succinate only’-energised condition and suggesting a transient peak in ROS can be efficient in pre-sensitising mPTP to Ca^2+^.

Despite Complex I-linked substrates generating higher mtROS at baseline, no increased sensitivity to Ca^2+^ -induced mPTP opening was observed. However, addition of rotenone under succinate-driven respiration reduced the probability of mPTP opening, suggesting that RET through complex I sensitises to mPTP opening, while forward electron flow through Complexes I and III does not affect the process. As expected, we observed an antimycin A-dependent increase in baseline mtROS production. Electron carriers upstream of the point of antimycin A-sensitive inhibition become reduced causing a large increase in baseline H_2_O_2_ production in mitochondria metabolising all substrates^48^. The antimycin A-dependent increase in H_2_O_2_ production is both greater after mPTP opening and sensitive to CsA and RuR, suggesting mPTP opening mediates the augmented mtROS production. Together, these observations suggest that complex III appears the major source of mtROS production following mPTP opening and, although glutamate/malate and succinate/rotenone produce ROS on complex III, it is not a sensitising factor for stimulating mPTP opening. ROS may have differential effects when generated at the inner or outer face of the mitochondrial inner membrane. Superoxide generation from complex I is directed towards the matrix and complex III to both the matrix and IMS, suggesting that complex I is the most significant source of superoxide generation towards the mitochondrial matrix^49, 50^. As superoxide does not cross membranes and is extremely reactive, it will initially react with components that are exposed in the environment next to the site of generation^51, 52^. Microdomains therefore are highly relevant for ROS species as they have a limited diffusion range due to their high, localised reactivity.

Under all metabolic conditions, rates of mitochondrial oxygen consumption increased following initial additions of Ca^2+^ preceding mPTP opening. Ca^2+^-induced stimulation of respiration has been proposed to occur through different mechanisms. Increased NADH production by Ca^2+^ sensitive dehydrogenases (pyruvate and oxoglutarate) of the TCA cycle^53^, Ca^2+^-mediated stimulation of succinate dehydrogenase^54^, or dissipation of the H^+^ gradient by futile Ca^2+^ cycling could augment electron flow through the respiratory chain^55^. However, under our conditions the Ca^2+^-induced respiratory increase was fully blocked both by CsA and RuR. Thus, we hypothesise that low-conductance, reversible mPTP opening, may be responsible for the respiratory increase observed following sub-threshold Ca^2+^ uptake^56, 57^. Given the absence of effect of low concentration Ca^2+^ on ΔΨ_m_, it is likely that these mild uncoupling events can be compensated for through increased respiration, as observed previously using FCCP at low concentration^47^. The lack of effect of Ca^2+^ on NADH provision to complex I may reflect that principal sites of NADH production using glutamate/malate are the Ca^2+^-insensitive glutamate and malate dehydrogenases and, additionally, that succinate dehydrogenase under these conditions has been shown to be inactivated by oxaloacetate^58, 59^.

High-conductance mPTP opening is consistent with mitochondrial inner membrane uncoupling and loss of ΔΨ_m_. In contrast to succinate-energised mitochondria, where respiration is maintained following mPTP opening, in glutamate/malate-energised mitochondria, respiratory rates decreased following Ca^2+^-induced high-conductance mPTP opening. Soluble NAD^+^/NADH is lost through the mPTP to the extra-mitochondrial space, limiting electron flux from UQ to complex III^60^. mPTP opening and respiratory decline were prevented by CsA and RuR as soluble reducing equivalent are preserved within the matrix. Intriguingly, addition of exogenous NADH or succinate following complete mPTP opening and inner membrane depolarisation is able to recover and increase respiration. These observations are consistent with those following Ca^2+^ overload and mPTP opening in succinate-driven respiration. The flavoprotein pool, via which electrons pass from succinate, is inner membrane bound, therefore facilitating maintenance of respiration as succinate saturates the pool and respiratory rate hits maximum. In contrast to respiration driven by glutamate/malate at complex I, where soluble respiratory intermediate NADH is lost from the matrix. Previously, Hawkins *et al*. observed that supplementation by succinate could prevent rotenone/oligomycin- and hypoxia-induced mitochondrial depolarisation. In agreement with the data presented here, succinate failed to maintain ΔΨ_m_ following mPTP opening^61^.

In summary, we investigated the role of substrate selectivity and utilisation in determining propensity for Ca^2+^-induced mPTP opening. We have characterised key modulatory factors, including mitochondrial energetic status and Ca^2+^ concentration in both regulating propensity for mPTP opening and determining consequence of pore opening. Together, these data further add to understanding the patho(physiological) effects of pore opening. For decades, mPTP opening has been recognised as a critical factor in cellular death across numerous diseases. Therefore, understanding the precipitating factors, the pathways culminating in pore opening and the downstream metabolic consequences will aid the rational design and development of therapeutics, with potential utility in treating multiple difficult-to-treat disease states.

## Materials and Methods

### Materials

All chemicals and compounds were purchased from Sigma-Aldrich (St. Louis, MO), unless otherwise specified. Fluorescent probes (tetramethylrhodamine methylester perchlorate; TMRM, Fluo-4FF, Amplex Red™; AmpR) were from Life Technologies (Eugene, OR).

### Animals

Female Sprague Dawley rats (250–300 g) were from Charles River (Wilmington, MA) and allowed to acclimatise to conditions for four days. Animals were euthanised by cervical dislocation followed by immediate removal of livers. Animal care and procedures were performed in accordance with UK Animals (Scientific Procedures) Act, 1986. Procedures were carried under a UK Home Office licence and studies were approved by Eisai’s Institutional Animal Care and Use Committee (IACUC).

### Isolation and storage of mitochondria

Mitochondria were isolated in accordance with published protocols^62^. Briefly, fresh rat livers were washed and finely minced in ice-cold wash buffer (250 mM sucrose, 10 mM KCl, 1 mM EGTA, 1 mM EDTA, 25 mM HEPES, adjusted to pH 7.5 using NaOH). Tissue was homogenised using 10 strokes of a glass/Teflon potter and drill, set to 1600 rpm, in 5x tissue volume of complete homogenisation buffer (300 mM trehalose dihydrate, 25 mM HEPES, 1 mM EGTA, 1 mM EDTA, 10 mM potassium chloride, 0.1% essentially fatty acid free bovine serum albumin (BSA) (Sigma, A3803), cOmplete Protease Inhibitor™ (Roche Diagnostics, Mannheim, Germany), adjusted to pH 7.5). Homogenates were centrifuged at 800 *g* for 10 minutes at 4 °C, supernatants transferred to a clean tube and then centrifuged further at 10,300 *g* at 4 °C for 10 minutes. Mitochondrial pellets were surface-washed using complete homogenisation buffer and the final centrifugation step repeated. The pellets were re-suspended in complete homogenisation buffer and protein concentration determined by bicinchoninic acid assay (BCA) (Thermo Scientific, Rockford, IL). Mitochondrial suspensions (50 mg protein ml^−1^) were snap-frozen in liquid nitrogen and stored at −80 °C until use. All mitochondrial preparations were maintained at −80 °C for up to 7 months. Prior to activity assays, frozen mitochondria were thawed by briefly placing vials in a 37 °C water bath and then kept on ice until required.

### Ca^2+^ retention capacity (CRC) assay using FLIPR^TETRA^

Assessment of Ca^2+^ retention capacity was used to assess *in vitro* sensitivity to Ca^2+^ of isolated mitochondrial preparations. Mitochondria were washed in ice-cold mitochondrial assay buffer (MAB; 75 mM mannitol, 25 mM sucrose, 5 mM potassium phosphate monobasic, 20 mM Tris base, 100 mM potassium chloride, 0.1% bovine serum albumin, adjusted to pH 7.4) to remove residual EDTA and re-suspended (2 mg protein ml^−1^, final assay concentration (FAC) = 1 mg protein ml^−1^) in complete MAB. To remove any contaminating Ca^2+^, MAB was pre-treated with Chelex 100 resin (Sigma-Aldrich, St. Louis, MO) and resin removed through filtration.

Complete MAB containing 2x Fluo-4FF penta-potassium salt (0.7 μM, FAC = 0.35 μM) was supplemented with either: (1) 20 mM L-glutamic acid, monosodium salt, FAC = 10 mM; 4 mM L-malic acid sodium salt, FAC = 2 mM, (2) 20 mM L-glutamic acid monosodium salt, FAC = 10 mM; 4 mM L-malic acid sodium salt, FAC = 2 mM; 6 mM NADH, FAC = 3 mM, (3) 20 mM succinate disodium salt, FAC = 10 mM or (4) 20 mM succinate disodium salt, FAC = 10 mM; 2 μM rotenone, FAC = 1 μM). Final pH of the solutions was confirmed to be 7.4 and adjusted where necessary using NaOH.

Mitochondrial suspensions (2x concentration; 20 μl) and supplemented Fluo-4FF containing MAB (2x concentration; 20 μl) were dispensed into a clear-bottom, black-walled 384 well plate containing compound using a Multidrop Combi Reagent Dispenser (Thermo Scientific, Rockford, IL) and incubated for 10 mins at room temperature. Extra-mitochondrial fluorescence (ex. 470–495/em. 515–575) was measured at 6 second intervals (FLIPR^TETRA^, Molecular Devices, Sunnyvale, CA) over 35 minutes at room temperature. CaCl_2_ (10 μM final concentration per addition) in MAB (2.5 μl additions to 40 μl) was repeatedly added at 3 minute intervals.

### Ca^2+^-induced mitochondrial membrane depolarisation using FLIPR^TETRA^

Mitochondrial ΔΨ_m_ was measured using tetramethylrhodamine methylester (TMRM), a voltage-sensitive cationic lipophilic dye, partitioning and accumulating in the mitochondrial matrix based upon the Nernst equation. When TMRM is loaded at relatively high concentrations (>100 nM^63^), fluorescence within the mitochondria is auto-quenched. Any disruption to mitochondrial function (e.g. membrane uncoupling or electron transport inhibition) dissipates ΔΨ_m_ with TMRM redistribution yielding an increase in fluorescence upon relief of auto-quenching.

To remove residual EDTA, mitochondria were centrifuged at 10,300 *g* at 4 °C for 10 minutes and re-suspended in ice-cold complete MAB to a final concentration of 2 mg protein ml^−1^ (final assay concentration (FAC) = 1 mg protein ml^−1^). MAB was pre-treated with Chelex 100 resin (Sigma-Aldrich, St. Louis, MO) to remove any contaminating Ca^2+^, and resin removed through filtration. MAB containing TMRM (2 μM, final assay concentration = 1 μM) was supplemented as for CRC assay. Mitochondrial suspension (2x concentration; 20 μl) and supplemented TMRM containing MAB (2x concentration; 20 μl) were dispensed into a clear-bottom, black-walled 384 well plate containing the compounds using a Multidrop Combi Reagent Dispenser (Thermo Scientific, Rockford, IL) and incubated with the compounds for 10 mins at room temperature to allow TMRM equilibration. Fluorescence (ex. 510–545/em. 565–625) was measured at 6 second intervals (FLIPR^TETRA^, Molecular Devices, Sunnyvale, CA) over 35 minutes at room temperature. CaCl_2_ (10 μM final concentration per addition) in MAB (2.5 μl additions to 40 μl) was repeatedly added at 3 minute intervals.

### Measurements of hydrogen peroxide generation using FLIPR^TETRA^

H_2_O_2_ production was determined using Amplex Red (AmpR), a dye reacting with 1:1 stoichiometry with H_2_O_2_ in a peroxidase-catalysed reaction to produce the highly fluorescent resorufin. Mitochondria were washed in ice-cold MAB to remove residual EDTA and re-suspended (2 mg protein ml^−1^, final assay concentration (FAC) = 1 mg protein ml^−1^) in complete MAB. MAB was pre-treated with Chelex 100 resin (Sigma-Aldrich, St. Louis, MO) to remove any contaminating Ca^2+^, and resin removed through filtration.

MAB containing 2x AmpR (20 μM) and horseradish peroxidase (HRP; 2 U ml^−1^) was supplemented as above for CRC assay. Mitochondrial suspension (2x concentration; 20 μl) and supplemented AmpR/HRP containing MAB (2x concentration; 20 μl) were dispensed into a clear-bottom, black-walled 384 well plate containing compound using a Multidrop Combi Reagent Dispenser (Thermo Scientific, Rockford, IL) and incubated with compound for 10 mins at room temperature. Fluorescence (ex. 510–545/em. 565–625) was measured at 6 second intervals (FLIPR^TETRA^, Molecular Devices, Sunnyvale, CA) over 35 minutes at room temperature. CaCl_2_ (10 μM final concentration per addition) in MAB (2.5 μl additions to 40 μl) was repeatedly added at 3 minute intervals.

### High-resolution respirometry and parallel fluorescence measurements

Mitochondrial oxygen consumption was measured using the Oxygraph 2 K (Oroboros Instruments, Innsbruck, Austria). Mitochondria were washed in ice-cold MAB, to remove residual EDTA. Mitochondria were re-suspended (1 mg protein ml^−1^) in MAB supplemented with either: (1) 10 mM L-glutamic acid, monosodium salt; 2 mM L-malic acid sodium salt, (2) 10 mM succinate disodium salt or (3) 10 mM succinate disodium salt; 1 μM rotenone. Mitochondrial suspensions were added to each chamber, maintained at 25 °C under constant stirring (250 rpm), Oxygraph 2 K calibrated using oxygen solubility factor 0.92 and respiration allowed to stabilise.

The Oxygraph 2 K was equipped with an O2K fluorimeter and fluorescence control unit (Oroboros Instruments, Innsbruck, Austria) and TMRM (2 μM), Fluo-4FF (1 μM) and Amplex Red/HRP (10 μM/1 U ml^−1^) were added to chambers for parallel measurement of ΔΨ_m_, extracellular Ca^2+^ and H_2_O_2_ respectively. A sequential titration protocol, including additions of 10 mM succinate disodium salt, 1 μM rotenone, 2.5 μM antimycin A, 0.5 μM carbonyl cyanide p-[trifluoromethoxy]-phenyl-hydrazone (FCCP) and 3 mM NADH disodium salt, 0.5 μM CsA and 0.5 μM RuR were added, as indicated in results. Extra-mitochondrial Ca^2+^ fluorescence (fluorescence-sensor blue; LED ex. 465), ΔΨ_m_ and H_2_O_2_ (both fluorescence-sensor green; LED ex. 525) were measured in parallel to respiration at 3 second intervals over the assay duration. CaCl_2_ (2.5 μM final concentration per addition) in MAB (2 μl additions to 2 ml) was repeatedly added at 1.5 minute intervals.

### Experimental design, data analysis and statistical procedures

Data are presented as mean ± standard deviation (s.d.). Normalisation of data allowed for control of inter-assay variability. AUC was calculated using ScreenWorks (Molecular Devices, Sunnyvale, CA). Curve fitting used GraphPad Prism version 7.02 for Windows (La Jolla, California, USA). Statistical significance tests performed are indicated in figure legends. Statistical significance was determined as *P* < 0.05.

## Electronic supplementary material

Supplementary Information
