# Supplementary material for: Mitochondrial permeability transition pore: sensitivity to opening and mechanistic dependence on substrate availability
Source: Sci Rep. 2017 Sep 5;7:10492. doi: 10.1038/s41598-017-10673-8 (PMC5585167; doi:10.1038/s41598-017-10673-8)
Supplement: Supplementary file 1 — Supplementary Information [file 41598_2017_10673_MOESM1_ESM.pdf]

Title: Mitochondrial permeability transition pore: sensitivity to opening and mechanistic dependence on substrate availability

Authors: Thomas Briston[1$], Malcolm Roberts[1], Sian Lewis[1], Ben Powney[1], James Staddon[1], Gyorgy Szabadkai[2, 3*], Michael R Duchen[2*]

Affiliations: [1] Neurology Innovation Centre, Hatfield Research Laboratories, Eisai Ltd., Hatfield, UK, [2] Department of Cell and Developmental Biology, Consortium for Mitochondrial Research, University College London, London, UK, [3] Department of Biomedical Sciences, University of Padua, Padua, Italy,

* Co-senior authors

Supplementary Figures

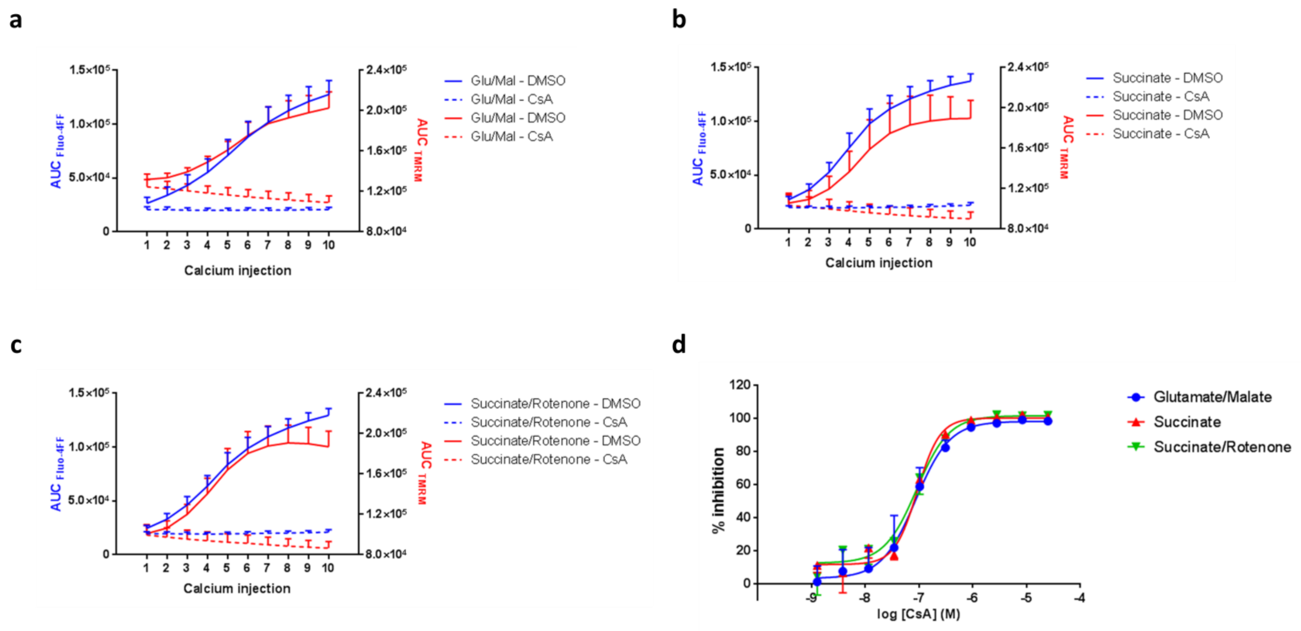

Supplementary figure 1: Effects of CsA on Ca^{2+}-induced mPTP opening. Mitochondria (1 mg protein ml^{-1}) were incubated with Fluo-4FF (0.35 µM) or TMRM (2 µM) in the presence of either (A) glutamate/malate (10 mM/2mM), (B) succinate (10 mM) or (C) succinate/rotenone (10 mM/1 µM), with or without CsA (1 µM; dashed trace). Pulses of $CaCl_2$ (10 µM) were added sequentially and

extra-mitochondria Ca^{2+} (Fluo-4FF; blue trace) and $\Delta\Psi_m$ (TMRM; red trace) recorded in parallel. Area under the curve was calculated between each $CaCl_2$ injection in mitochondrial energised using defined substrates. (D) Mitochondria were incubated as above with Fluo-4FF and subject to a Ca^{2+} retention capacity protocol. Area under the curve was calculated between $CaCl_2$injections 5-6 and results are expressed as % inhibition, normalised to DMSO (0% inhibition) and CsA (5 µM; 100% inhibition). Data are expressed as means (± s.d.) of at least three independent experiments. Curves were fitted using 4-parameter logistic equation (GraphPad Prism). Abbreviations: CsA; cyclosporin A, TMRM; tetramethylrhodamine methylester, Glu; glutamate, Mal; malate, Succ; succinate, AUC; area under the curve.

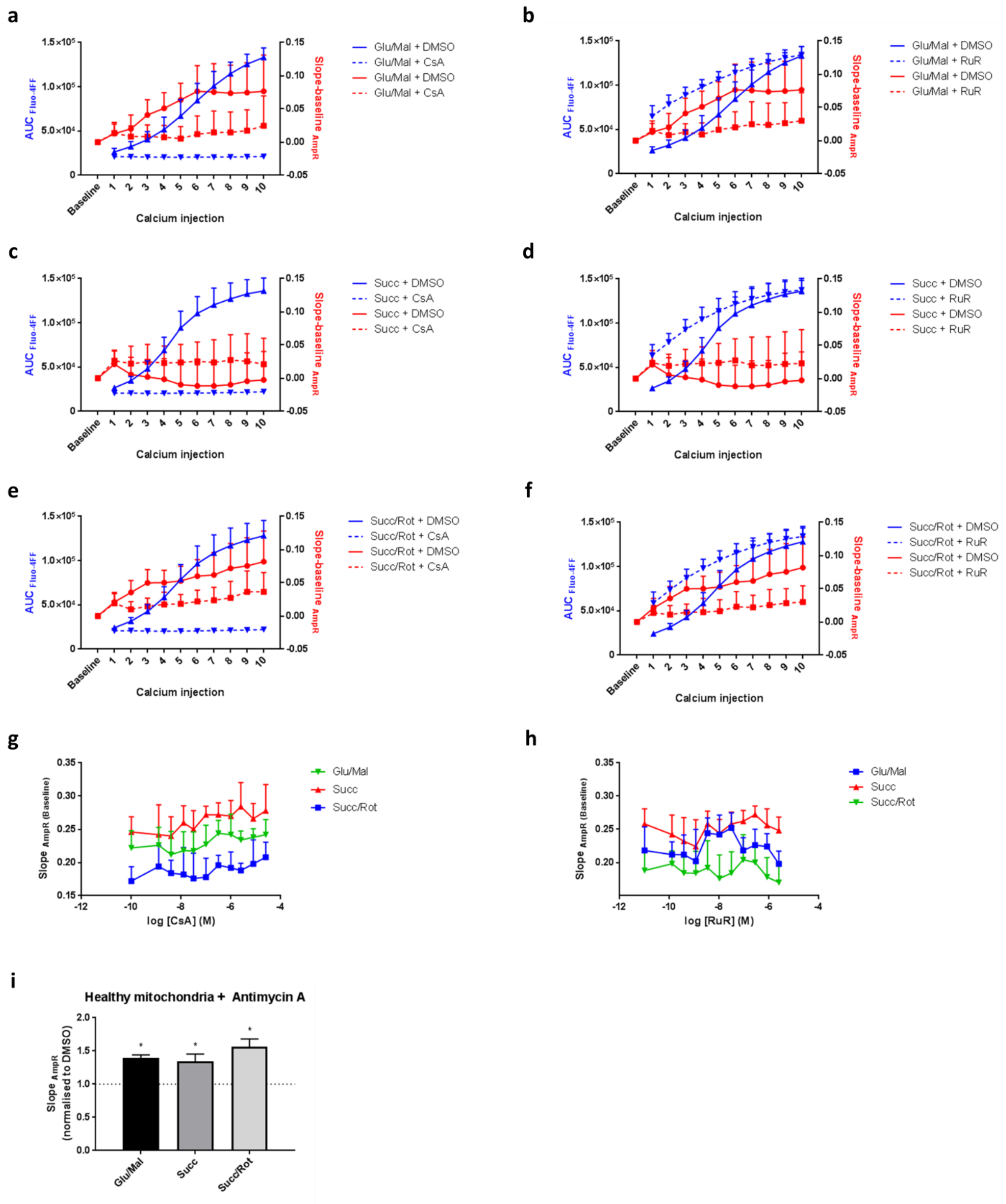

Supplementary figure 2: Effects of CsA and RuR on Ca^{2+}-induced H_2O_2 production. Mitochondria (1 mg protein ml^{-1}) were incubated with Fluo-4FF (0.35 µM) or AmpR/HRP (10 µM/1 U ml^{-1}) in the presence of either glutamate/malate (10 mM/2mM), succinate (10 mM) or succinate/rotenone (10 mM/1 µM). Pulses of $CaCl_2$ (10 µM) were added sequentially and extra-mitochondria Ca^{2+} (Fluo-4FF; solid trace) and H_2O_2 (AmpR; dashed trace) recorded in parallel. Area under the curve (Fluo-4FF) and

slope (AmpR; normalised for baseline) were calculated between each $CaCl_2$ injection in mitochondrial energised using defined substrates. Mitochondria were incubated in presence of (A, C, E) CsA (1 µM) or (B, D, F) RuR (1 µM) and extra-mitochondrial Ca^{2+} and H_2O_2 production was measured in parallel. Abbreviations: CsA; cyclosporin A, RuR; ruthenium red, AmpR; amplex red, Glu; glutamate, Mal; malate, Succ; succinate, Rot; rotenone, AUC; area under the curve. (G) Mitochondria (1 mg protein ml^{-1}) were incubated with AmpR/HRP (10 µM/1 U ml^{-1}) in the presence of either glutamate/malate (10 mM/2mM), succinate (10 mM) or succinate/rotenone (10 mM/1 µM). Mitochondria were incubated in presence of (D) CsA and (E) RuR in a 2-fold dilution series under different metabolic conditions. H_2O_2 production was measured prior to $CaCl_2$ injection to identify compound-induced baseline changes in mtROS. Data is presented as change in AmpR fluorescence over time (slope). (I) Mitochondria (1 mg protein ml^{-1}) were incubated with AmpR/HRP (10 µM/1 U ml^{-1}) in the presence of either glutamate/malate (10 mM/2mM), succinate (10 mM) or succinate/rotenone (10 mM/1 µM). Antimycin A (2.5 µM) was added for 10 minutes and fluorescence measured. Data is presented as change in AmpR fluorescence over time (slope) normalised to data in the presence of DMSO alone. Data was analysed using one-way ANOVA, corrected for multiple comparisons using Holm-Sidak method. No symbol $P > 0.05$, * $P < 0.0001$.
